# miR-23a-3p and miR-181a-5p modulate SNAP-25 expression

**DOI:** 10.1371/journal.pone.0279961

**Published:** 2023-01-17

**Authors:** Simone Agostini, Elisabetta Bolognesi, Roberta Mancuso, Ivana Marventano, Lorenzo Agostino Citterio, Franca Rosa Guerini, Mario Clerici

**Affiliations:** 1 IRCCS Fondazione Don Carlo Gnocchi ONLUS, Milan, Italy; 2 Department of Pathophysiology and Transplantation, University of Milan, Milan, Italy; Nippon Medical School, JAPAN

## Abstract

SNAP-25 protein is a key protein of the SNARE complex that is involved in synaptic vesicles fusion with plasma membranes and neurotransmitter release, playing a fundamental role in neural plasticity. Recently the concentration of three specific miRNAs–miR-27b-3p, miR-181a-5p and miR-23a-3p –was found to be associated with a specific *SNAP-25* polymorphism (rs363050). *in silico* analysis showed that all the three miRNAs target *SNAP-25*, but the effect of the interaction between these miRNAs and the 3’UTR of *SNAP-25* mRNA is currently unknown. For this reason, we verified *in vitro* whether miR-27b-3p, miR-181a-5p and miR-23a-3p modulate SNAP-25 gene and protein expression. Initial experiments using miRNAs-co-transfected Vero cells and *SNAP-25* 3’UTR luciferase reporter plasmids showed that miR-181a-5p (p≤0.01) and miR-23a-3p (p<0.05), but not miR-27b-3p, modulate the luciferase signal, indicating that these two miRNAs bind the *SNAP-25* 3’UTR. Results obtained using human oligodendroglial cell line (MO3.13) transfected with miR-181a-5p or miR-27b-3p confirmed that miR-181a-5p and miR-23a-3p regulate SNAP-25 gene and protein expression. Interestingly, the two miRNAs modulate in an opposite way SNAP-25, as miR-181a-5p significantly increases (p<0.0005), whereas miR-23a-3p decreases (p<0.0005) its expression. These results for the first time describe the ability of miR-181a-5p and miR-23a-3p to modulate SNAP-25 expression, suggesting their possible use as biomarkers or as therapeutical targets for diseases in which SNAP-25 expression is altered.

## Introduction

Soluble N-ethylmaleimide-sensitive factor attachment protein receptor (SNARE) is a complex that mediates pore formation and vesicle fusion and plays a key role in neural plasticity [1, 2]. Three specific proteins assemble this complex: Syntaxin 1 (STX1), synaptosomal-associated protein of 25 KDa (SNAP-25) and vesicle associated membrane protein 2 (VAMP2); these three proteins, together with others e.g. synapsins, synaptophysin and proteins of SH3 And Multiple Ankryn Repeat Domain families (SHANK), are vitally important in the mediation of synaptic communication [3–5].

Dysfunction of the SNARE complex and de-regulation of its constitutive proteins are associated to several diseases [6–8]. In particular, *SNAP-25* polymorphisms, gene expression, and protein concentration, have been associated with different neurological and non-neurological diseases, including Alzheimer’s disease (AD) [9–12], Parkinson’s disease (PD) [13], neurodevelopmental disorders such as Autism and Borderline Intellectual Functioning [14–16], Attention Deficit and Hyperactivity Disorder (ADHD) [17], type 2 diabetes [18, 19] and sarcopenia [20]. Notably, SNAP-25 protein expression was shown to be reduced in brains of AD [21, 22], whereas it was observed to be increased in cerebrospinal fluids of AD [23] and PD patients [24].

The *SNAP-25* gene (88,591 nucleotides) is located on chromosome 20 (20p12.2), and codes for a 206 amino acids (aa) protein, formed by three different domains: a N-terminal α-helix domain, a palmitoylation cysteine-rich domain, and a C-terminal α-helix domain^1^. Molecular mechanisms for SNAP-25 expression are not fully understood; microRNAs, by binding the 3’ untranslated region (3’UTR) of mRNA target [25, 26], can contribute to post-transcriptional modulation of the expression of SNAP-25, resulting in disease associated misregulation of this protein. Several works have shed light on the role played by miRNAs on the regulating key synaptic components; results [27] convincingly showed that the expression of these proteins is modulated by the interactions, between, e.g. STX1 and miR-29a [28], SNAP-25, miR-153 and miR-27 [29, 30], TXN2 (thioredoxin) and miR-27 [30] and, finally, SHANK3 (SH3 And Multiple Ankyrin Repeat Domain 3) and miR-34a [31].

We have previously shown that 3 miRNAs (miR-27b-3p, miR-23a-3p and miR-181a-5p) targeting SNAP-25 3’UTR are differentially expressed in serum of AD patients in relation with *SNAP-25* rs363050 polymorphism. Thus, the concentration of these proteins was significantly reduced in AD patients carrying the *SNAP-25* rs363050 GG genotype compared with those carrying the AA or AG genotypes, and was also greatly diminished when the AD patients were compared to subjects with Mild Cognitive Impairment (MCI) or healthy controls [32]. The effects of these miRNAs on SNAP-25 gene and protein expression have nevertheless never been examined; we decided to focus our efforts on clarifying this topic.

## Material and methods

### Cell lines

The MO3.13 immature (non-differentiated) human oligodendrocyte cell line (a 2005 kind gift from Dr. Neil Cashman, University of Toronto, Canada) [33, 34] and the Vero African green monkey kidney cell line (ATCC CCL-81, purchased from ATCC) were used in this study. The MO3.13 cell line was derived from humans with no history of clinical signs of infectious diseases, and the cell line has shown no signs of contamination, including cytopathic effects.

All cell lines were cultured in Dulbecco’s Modified Eagle Medium (DMEM, PAN-Biotech GmbH, Aidenbach, Germany) supplemented with 10% fetal bovine serum (FBS, PAN-Biotech), 50 U/ml penicillin and 50 μg/ml streptomycin (Euroclone, Rho, Milan, Italy), and maintained in an incubator at 37°C with 5% CO_2_. Trypan blue exclusion test was used to evaluate cell viability, with an automatic cell counter (TC20 Automatic Cell Counter, BioRad, Hercules, CA, US). To note, as previously described in a recent paper, the MO3.13 remained undifferentiated throughout the transfection experiments (at least 72 hours) [35].

### Vero cells: Transfection and luciferase assay

Vero cells were seeded at 10^4^ cells/well into a 96-well plate and co-transfected with a luciferase reporter plasmid containing the 3’UTR of SNAP-25 mRNA (Prod. ID: S808485, Active Motif, Carlsbad, CA, US) (genomic coordinates: chromosome 20; 10,226,822–10,228,152), together with mimic, inhibitor miRNAs (miR23a-3p, miR27b-3p and miR-181a-5p), or scramble: *C*. *el* miR-39-3p, Qiagen GmbH, Hilden, Germany) (2 μM), using the DharmaFECT Duo Transfection (SwitchGear Genomics, Carlsbad, CA, US), according to manufacturers’ instruction. The same experiments were also performed using an empty 3’UTR vector (negative control). The concentration of the plasmids was 20 μg/ml. After the co-transfection, cells were incubated in a humidified 5% CO_2_ incubator at 37°C for 24 hours.

A luciferase activity was assessed using the LightSwitch Luciferase Reagent (SwitchGear Genomics), as previously reported [35].

### MO3.13 cells: Transfection

Plated in 6-well plates at a density of 6 x 10^5^ cells/well, MO3.13 cells were transfected, separately, with scramble miRNA and the selected mimic or inhibitor miRNAs (Qiagen GmbH, Hilden, Germany) (50 nM). The transfection was performed by Lipofectamine RNAiMAX reagent (Life Technologies, Foster City, CA, US), according the manufacturer’s instruction. After transfection, the cells were incubated at 37°C for 6, 24, 48 and 72 h in a humidified 5% CO_2_ incubator. Each experiment was repeated at least 3 times and conducted in triplicate.

### MO3.13 cells: RNA extraction and mRNA SNAP-25 quantitation by ddPCR and qPCR

Total RNA, extracted from the transfected MO3.13 cells using a column-based kit (QIAamp RNA Blood, Qiagen, Hilden, Germany), as previously reported [35], was retrotranscribed with High-capacity cDNA Reverse Transcription kit (Life Technologies), as specified by the manufactures.

One ng of cDNA was used for the quantification of *SNAP-25* gene expression (specific assay by Life Technologies) by digital droplet PCR (ddPCR QX200, Bio-Rad, Hercules, CA, US). Absolute *SNAP-25* gene expression was reported as copies/ng RNA. For the methodologies of ddPCR, see our previous article [20]. *SNAP-25* gene expression was also evaluated by quantitative PCR (qPCR, CFX Touch real-time PCR, Bio-Rad, Hercules, CA, US). Specific TaqMan gene expression assays (Life Technologies, Foster City, CA, US) were utilized to detect the expression of *SNAP-25* (ID: Hs00938957_m1) and of the *YWHAZ* reference gene (ID: Hs03044281_g1). Each cDNA template was tested in triplicate, with non-template control for each session.

### MO3.13 cells: SNAP-25 protein quantitation

MO3.13 cells were mechanically disrupted by 3 repeated cycles of freezing and thawing. SNAP-25 protein was measured in MO3.13 cellular lysate (diluted 1:10) by ELISA, according to the manufacturer’s instruction (LSBio, LifeSpan BioSciences, Inc, Seattle, WA, US). The optical densities (OD) for each well were determined at 450 nm by plate reader (Sunrise, Tecan, Mannedorf, Switzerland), and optical densities (OD) were determined at 450 nm. SNAP-25 concentration was expressed as ng/mL (sensitivity: 0.78 ng/mL).

### MO3.13 cells: SNAP-25 immunofluorescent staining by flow cytometry

To quantify the transmembrane SNAP-25 protein by flow cytometry, MO3.13 cells were treated with trypsin 10% for 5 minutes, collected and washed in phosphate-buffered saline (PBS) for 10 minutes at 1500 g. Cells were resuspended in 100 μl of PBS, and stained with mouse IgG1 monoclonal anti-human antibody SNAP-25 FITC (clone SP12, ThermoFisher Scientific, Rockford, IL, US). Cells were finally incubated for 30 minutes at room temperature and washed for 10 minutes at 1500 g. Analyses were performed using a Beckman-Coulter GALLIOS flow cytometer equipped with a 22 mW Blue Solid State Diode laser operating at 488 nm and with a 25 mW Red Solid State Diode laser operating at 638 nm and interfaced with Kaluza analysis software. Two-hundred-thousand events were acquired and gated on Forward and Side scatter properties. Samples were first run using mouse IgG1 isotype control (labeled FITC, code X0927, Dako, Agilent, Santa Clara, CA, US), and the mean fluorescence intensity (MFI) was calculated on MFI-positive cells alone.

### Statistical analyses

Data were normally distributed and are presented as mean ± standard deviation (SD). Two-way ANOVA and, when appropriate, the Student’s t-test were used to analyze data. QuantaSoft software version 1.7.4.0917 (Bio-Rad) was used to quantify the results of ddPCR for the gene expression of *SNAP-25*. Threshold were determined manually for each experiment, according to the negative controls, which included a no template control. Droplet positivity was determined by fluorescence intensity; only droplets above a minimum amplitude threshold were counted as positive. For qPCR analysis, gene expression was presented as the normalization ratio calculated as follows: fold = 2^-[ΔCq(sample) - ΔCq(scramble)]^ [36].

## Results

### Luciferase assay

To verify the binding of the three analyzed miRNAs–miR-23a-3p, miR-27b-3p and miR-181a-5p **–**to SNAP-25 mRNA 3’UTR, luciferase activity in cells lysate of Vero cells that had been co-transfected with these miRNAs was measured. Transfected Vero cells vitality was over 95%, without any toxic effect of the miRNAs and plasmid. The relative luciferase activity (RLA) was measured 24 hours after co-transfection.

**Fig 1:**
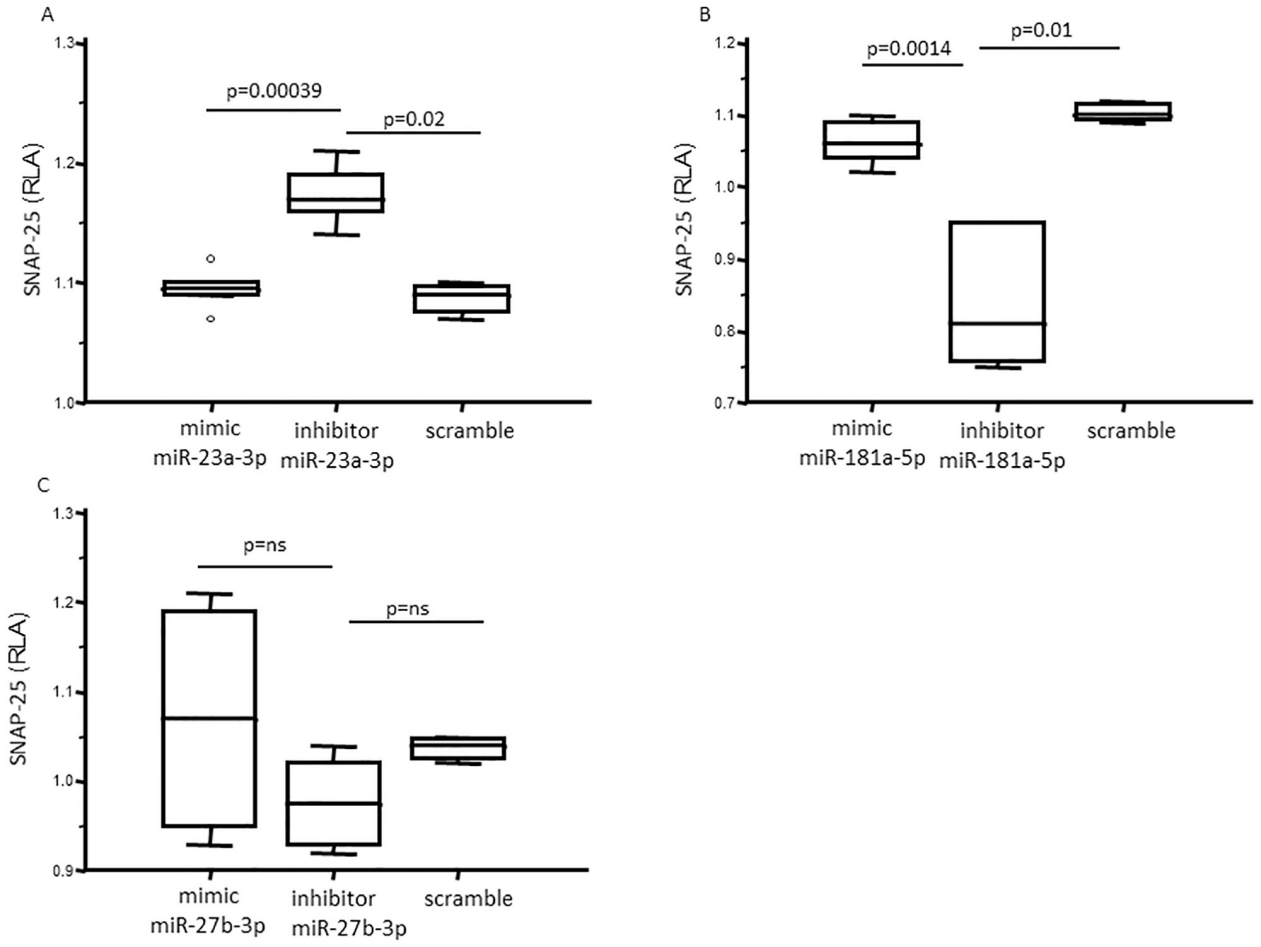
Relative luciferase activity (RLA) in Vero cells. Different transfection experiments were performed independently with miRNA mimic, or miRNA inhibitor or scramble control. Each experiment was repeated at least 3 times and conducted in triplicate; the results presented in the figures indicate the median values obtained and 25^th^-75^th^ percentile. RLA were measured in Vero cells 24 hours after transfection with (a) miR-23a-3p mimic, or inhibitor, or scramble negative control; (b) miR-181a-5p mimic, or inhibitor or scramble negative control; (c) miR-27b-3p mimic, or inhibitor or scramble negative control. RLA of cells transfected with miR-23a-3p inhibitor was significantly higher compared to that observed in cells transfected with miR-23a-3p mimic or scramble negative control; RLA of cells transfected with miR-181a-5p was significantly lower compared to that observed in cells transfected with miR-181a-5p mimic or scramble negative control. No differences were observed regarding miR-27b-3p mimic or inhibitor transfection. ns: not significative.

Regarding miR-23a-3p, RLA was differentially measured among Vero cells co-transfected with mimic, inhibitor and scramble (p = 0.005). In particular, the RLA of inhibitor miR23a-3p co-transfected Vero cells was significantly higher (RLA: 1.17±0.05) compared to that observed in cells co-transfected with either scramble miRNA (RLA: 1.08±0.02, p = 0.02) or mimic miR23a-3p (1.09±0.02, p = 0.004); no statistical differences were observed between Vero cells co-transfected with mimic and scramble (Fig 1(A)). Similar results were obtained with miR-181a-5p. Thus, RLA was differentially measured among Vero cells that had been co-transfected with mimic, inhibitor and scramble (p = 0.004). However, in this case the RLA of inhibitor miR-181a-5p co-transfected Vero cells was significantly lower (RLA: 0.84±0.09) than those observed in cells co-transfected with either scramble miRNA (RLA: 1.09±0.02, p = 0.01) or mimic miR-181a-5p (RLA: 1.06±0.03, p = 0.0014), whereas, again, no statistical differences were observed between Vero cells co-transfected with mimic and scramble (Fig 1(B)). Finally, no differences were observed when RLA of mimic, inhibitor and scramble miR-27b-3p-co-transfected Vero cells was analyzed (Fig 1(C)).

Taken together these results suggest that mi-23a-3p and miR-181a-5p, but not miR-27b-3p, bind the 3’UTR of SNAP-25 and, as a result, interfere with luciferase expression.

### MO3.13 cells: SNAP-25 mRNA and protein quantitation

Because the luciferase experiment showed that only miR-23a-3p and miR-181a-5p modulate SNAP-25, we focused on these two miRNAs alone and examined their activity using human immature oligodendrocyte cells, MO3.13. Also in this case, cell vitality was over 95% in every transfection experiment.

**Fig 2:**
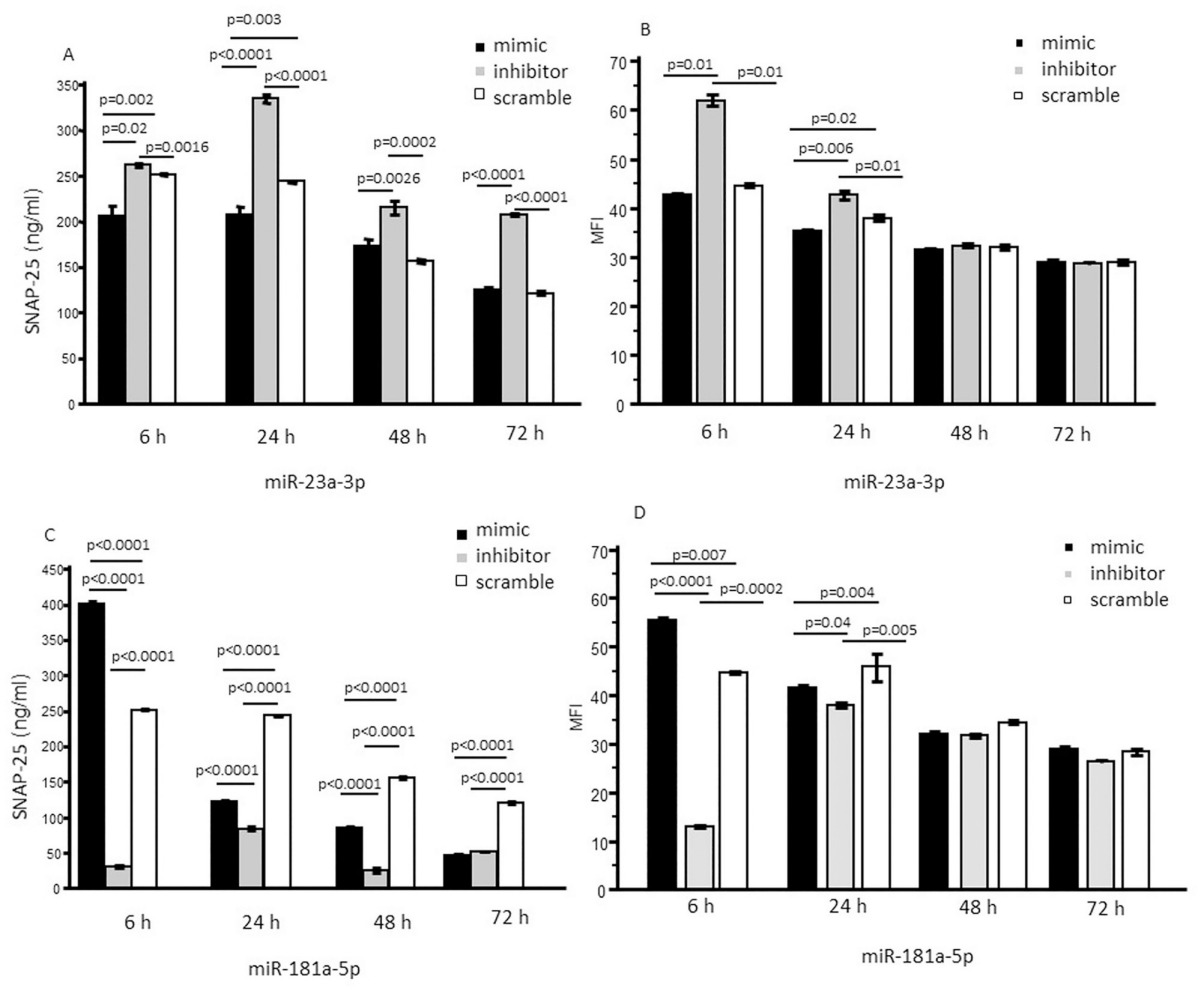
SNAP-25 protein concentration in MO3.13. Different transfection experiments were performed independently with miRNA mimic, or miRNA inhibitor or scramble control. Each experiment was repeated at least 3 times and conducted in triplicate; the results presented in the figures indicate the mean values obtained. SNAP-25 protein concentration (ng/ml) were measured in MO3.13 cells in different time points: 6, 24, 48 and 72 hours after transfection with (a) miR-23a-3p mimic, or inhibitor or scramble negative control by ELISA (a) and by flow cytometry (b); miR-181a-5p mimic, or inhibitor or scramble negative control by ELISA (c) and by flow cytometry (d). SNAP-25 concentration in cells transfected with miR-23a-3p inhibitor was significantly higher compared to that observed in cells transfected with miR-23a-3p mimic or scramble negative control in each time point; SNAP-25 concentration in cells transfected with miR-181a-5p inhibitor was significantly lower compared to that observed in cells transfected with miR-181a-5p mimic or scramble negative control in each time point.

Results showed that, upon miR-23a-3p inhibitor transfection, SNAP-25 production by MO3.13 was significantly increased at each time-point (6, 24, 48 and 72 hours post transfection) (p<0.005 for each time point) compared to what was observed in scramble-transfected cells. An opposite effect was detected when SNAP-25 protein production was analyzed in miR-23a-3p mimic-transfected MO3.13 cells. In this case SNAP-25 production was significantly reduced compared to scramble-transfected MO3.13 cells, 6 and 24 hours post transfection. Notably, SNAP-25 generation returned to normal 72 hours post transfection (Fig 2(A)). Immunostaining analysis by flow cytometry showed comparable effects for mimic and inhibitory transfection experiments on SNAP-25 protein expression. In particular, the miR-23a-3p inhibitor significantly increased SNAP-25 production as compared to what was observed in cells that had been transfected by the scrambled miRNA 6 and 24 hours post transfection (p<0.05). On the converse, transfection with miR-23a-3p significantly down-regulated SNAP-25 production compared to scramble miRNA transfected cells 24 hours post transfection (p = 0.02) (Fig 2(B)).

**Fig 3:**
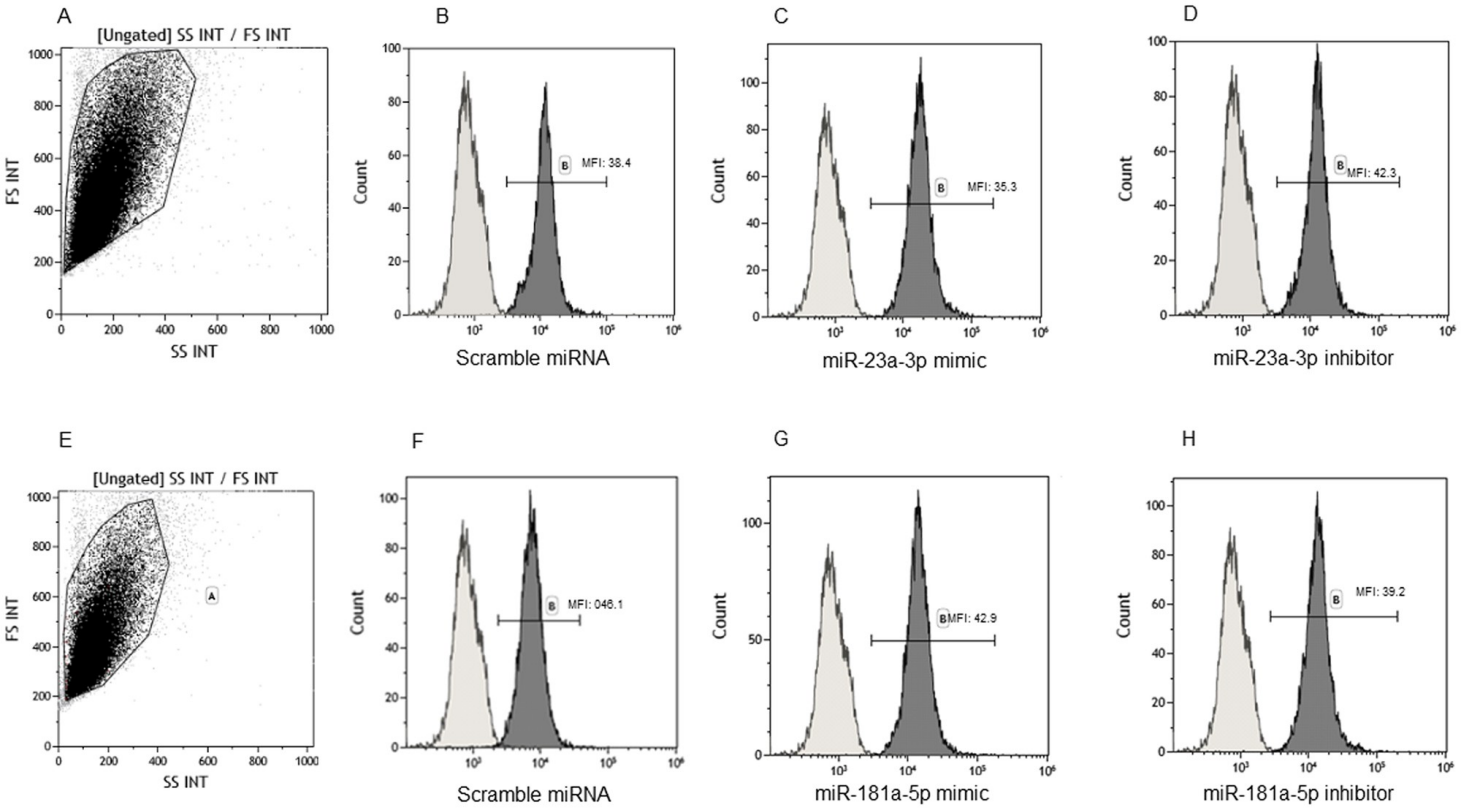
SNAP-25 immunostaining in MO3.13. Representative results obtained by staining MO3.13 with mAbs specific for SNAP-25. Gate on forward and side scatter plot (a and e) and (in dark grey diagrams) SNAP-25 signal intensity of MO3.13 cells transfected with scramble miRNA (b and f), miR-23a-3p mimic (c), or inhibitor (d), and miR-181a-5p mimic (g), or inhibitor (h). Light grey diagrams in b, c, d, f, g and h represent the isotype control. MFI: mean fluorescence intensity.

On the converse, SNAP-25 protein production was significantly lower at each time-point (6, 24, 48 and 72 hours post transfection) (p<0.0001 for each time point) in MO3.13 cells transfected with miR-181a-5p inhibitor compared to those transfected with scramble miRNA. Finally, the concentration of this protein was significantly augmented in MO3.13 cells transfected with the mimic compared to those transfected with scramble at 6 hours post transfection (p<0.05) (Fig 2(C)). Flow cytometry analysis showed comparable effects for mimic and inhibitory transfection experiments. In particular, SNAP-25 expression was significantly reduced at 6 and 24 hours post transfection in MO3.13 cells by the miR-23a-3p inhibitor, whereas MO3.13 cells transfected by the mimic miRNA were characterized by a significantly increased production of SNAP-25 compared to those transfected by scramble miRNA (p<0.05) (Fig 2(D)). Representative dot plot and histograms are shown in Fig 3.

No statistical differences were observed when *SNAP-25* gene expression was investigated (Table 1) by ddPCR; notably, at 6 hours post transfection miR-181a-5p mimic transfection SNAP-25 mRNA concentration was much higher compared to all the other conditions, analogously to what was observed when protein production was analyzed. Comparable results were obtained with qPCR analysis (S1 Table). Importantly, data obtained using ddPCR and qPCR significantly and positively correlated with each other (p = 0.02).

**Table 1:** *SNAP-25* gene expression (copies/ng) in MO3.13 cells transfected with miR-23a-3p mimic, miR23a-3p inhibitor, miR-181a-5p mimic, miR-181a-5p inhibitor, and scramble negative miRNA. The *SNAP-25* expression was measured in different time points: 6 hours after transfection, 24 hours after transfection, 48 hours after transfection, and 72 hours after transfection. Data are expressed as mean ± standard deviation.

Timing	Transfection	SNAP-25 mRNA (copies/ng)
6 hours after transfection	scramble miRNA	14.31±2.88
	miRNA-23a-3p mimic	21.65±10.34
	miRNA-23a-3p inhibitor	19.36±7.35
	miRNA-181a-5p mimic	57.42±22.60
	miRNA-181a-5p inhibitor	21.45±16.32
24 hours after transfection	scramble miRNA	13.75±7.15
	miRNA-23a-3p mimic	23.36±10.26
	miRNA-23a-3p inhibitor	22.55±5.42
	miRNA-181a-5p mimic	9.26±4.83
	miRNA-181a-5p inhibitor	7.24±4.21
48 hours after transfection	scramble miRNA	3.24±1.14
	miRNA-23a-3p mimic	3.56±0.12
	miRNA-23a-3p inhibitor	0.65±0.02
	miRNA-181a-5p mimic	3.13±0.22
	miRNA-181a-5p inhibitor	4.22±1.36
72 hours after transfection	scramble miRNA	2.12±0.13
	miRNA-23a-3p mimic	3.76±0.01
	miRNA-23a-3p inhibitor	0.72±0.32
	miRNA-181a-5p mimic	2.24±0.15
	miRNA-181a-5p inhibitor	2.25±0.91

These results confirm that miR-23a-3p and miR-181a-5p interact with 3’UTR SNAP-25, and demonstrate that the effect of such interaction is diametrically different, as miR-23a-3p down-regulates whereas miR-181a-5p up-regulates SNAP-25 protein and gene activity.

## Discussion

The aim of the present study was to verify whether miR-23a-3p, miR-181a-5p and miR-27b-3p can modulate the expression of SNAP-25, as *in silico* they are able to bind its 3’UTR. It is very important to verify this interaction: it is known that SNAP-25 is involved in Alzheimer’s disease [5], and also these three miRNAs seem to be associated with the disease [32, 37–39]. Moreover, miR-23a-3p and miR181b-5p were shown to be associated as well with Autism Syndrome Disorder (ASD) [40–43], a disease in which even SNAP-25 is also suggested to play a role. In particular, in ASD children *SNAP-25* polymorphisms were shown to be associated with hyperactivity and cognitive impairment [14, 15], leading to the hypothesis that the functioning of synapses related with cognitive activity are influenced by different expression of SNAP-25 protein [15]. The real nature of the interaction between these SNPs and SNAP-25, as well as the possible functional consequences of such interaction, though, have not yet been analyzed.

To address this question, we initially used a non-human cell line–Vero cells–that as per definition does not express the human SNAP-25. These cells were co-transfected independently with the three selected miRNAs and with a plasmid containing the 3’UTR of human SNAP-25 linked with luciferase, so that the luciferase activity was subordinated by the potential binding between the 3’UTR and miRNA. Results showed that miR-23a-3p and miR-181a-5p modulate the luciferase activity, and have an opposite effect on such activity, thus miR-181a-5p enhances and miR-23a-3p reduces the expression of luciferase. In contrast with the previous results, miR-27b-3p, had no effect on luciferase activity, indicating that that this miRNA, which *in silico* is theoretically able to interact with the SNAP-25 3’UTR, actually cannot bind this genetic region. To note, in a previous work, Machitani and coworkers [30], found that pre-miR-27b is able to suppress the SNAP-25 expression: as they performed transfection experiments with a pre-miRNA, and on the base of our results, it is plausible that their results are due to miR-27b-5p and not to miR-27b-3p. Next, on the basis of these results, we focused our attention of miR-23a-3p and miR-181a-5p, to verify whether these two miRNAs can modulate the SNAP-25 expression in human oligodendrocyte cells. To this end we used the MO3.13 cell line, a cell line that expresses SNAP-25. Transfection of MO3.13 cells with the appropriate mimic and inhibitor miRNAs were performed, and SNAP-25 mRNA and protein were quantified. Results confirmed that miR-181a-5p increases whereas miR-23a-3p decreases SNAP-25 protein expression. This effect was very clear and significant for protein concentration, whereas results for mRNA expression were not as compelling. However, this is not an unexpected result, as miRNA-mediated gene silencing is a complex mechanism in which several and redundant mechanisms are involved [44]. Moreover, the predominant mechanism of direct miRNAs action is translational inhibition [45], as several studies found that miRNAs can inhibit protein synthesis without affecting mRNA levels [46, 47], possibly because of the presence of alternate isoforms, post-translational modification or other factors involved in the silencing. The results regarding the miR-181a-5p capacity to increase the SNAP-25 expression is quite unexpected, as usually miRNAs act as suppressor of gene expression: future studies are required to address this issue.

miR-23a-3p is localized on chromosome 19 (19p13.12), and was identified for the first time in 2001 [48]. miR-23a-3p is a little-known miRNA, mainly found to be deregulated in cancers [49, 50]. However, it is important to underline that this miRNA was found to be expressed in synapses in animal models [51]. Additional results indicated that miR-23a-3p expression increases in isolated neuropiles after long-term potentiation, a synaptic plasticity form whereby a long-lasting enhancement of synaptic transmission is due to high-frequency stimulation [52]. Because SNAP-25 is a synaptic protein, these findings support our results, and it is plausible that the results obtained in rats could be the consequence of the binding between SNAP-25 and miR-23a-3p.

miR-181a-5p is localized on chromosome 1 (1q32.1), and was identified in human cells for the first time in 2003 [53]. Also this miRNA is barely known, although it seems to be involved in cell proliferation and differentiation in cancer [54], and as regulator of inflammation in macrophages and dendritic cells [55]. Importantly, recent studies showed that this miRNA is expressed in neurons, where it could have a role in synaptic development, function and plasticity [56, 57], possibly *via* SNAP-25 interaction.

Taken together, although they will need to be further confirmed by other analyses, results herein for the first time strongly suggest that miR-23a-3p and miR-181a-5p interact with the *SNAP-25* gene acting as modulators of protein expression and having an opposite role on such expression. A limitation of our study is the lacking of immunoblotting and immunostaining experiments to confirm the exact relation between miRNAs and SNAP-25.

It remains to be clarified whether these miRNAs bind SNAP-25 mRNA directly or by interacting with other molecular factors, why they have an opposite effect on gene expression and if they have an effect also on other SNARE complex proteins. Because both SNAP-25 and miR-23a-3p and miR-181a-5p were found associated with Alzheimer’s disease, Mild Cognitive Impairment and ASD, the combined study of these biological parameters could yeld new important information regarding the mechanisms underlying these conditions. Finally, results also suggest that these miRNAs could be considered as therapeutical target in diseases in which SNAP-25 is involved, and that the analysis of their concentration in biological samples could offer a novel diagnostic and prognostic tool.

## Supporting information

S1 Table *SNAP-25* gene expression (fold) in MO3.13 cells transfected with miR-23a-3p mimic, miR23a-3p inhibitor, miR-181a-5p mimic, miR-181a-5p inhibitor. The *SNAP-25* expression was measured in different time points by qPCR: 6 hours after transfection, 24 hours after transfection, 48 hours after transfection and 72 hours after transfection. Data are expressed as mean ± standard deviation.
(DOCX)
