# Supplementary material for: miR-23a-3p and miR-181a-5p modulate SNAP-25 expression
Source: PLoS One. 2023 Jan 17;18(1):e0279961. doi: 10.1371/journal.pone.0279961 (PMC9844927; doi:10.1371/journal.pone.0279961)
Supplement: S1 Table — The SNAP-25 expression was measured in different time points by qPCR: 6 hours after transfection, 24 hours after transfection, 48 hours after transfection and 72 hours after transfection. Data are expressed as mean ± standard deviation. (DOCX) [file pone.0279961.s001.docx]

**Supplementary Table 1.** *SNAP-25* gene expression (fold) in MO3.13 cells transfected with miR-23a-3p mimic, miR23a-3p inhibitor, miR-181a-5p mimic, miR-181a-5p inhibitor. The *SNAP-25* expression was measured in different time points by qPCR: 6 hours after transfection, 24 hours after transfection, 48 hours after transfection and 72 hours after transfection. Data are expressed as mean ± standard deviation.

| **Timing** | **Transfection** | **SNAP-25 mRNA (fold)** |
| --- | --- | --- |
|  | miRNA-23a-3p mimic | 1.31±0.07 |
| 6 hours after transfection | miRNA-23a-3p inhibitor  miRNA-181a-5p mimic  miRNA-181a-5p inhibitor | 1.36±0.25  2.23±0.48  1.12±0.32 |
|  | miRNA-23a-3p mimic | 1.22±0.12 |
| 24 hours after transfection | miRNA-23a-3p inhibitor  miRNA-181a-5p mimic  miRNA-181a-5p inhibitor | 1.26±0.25  0.89±0.09  0.85±0.33 |
|  | miRNA-23a-3p mimic | 1.39±0.10 |
| 48 hours after transfection | miRNA-23a-3p inhibitor  miRNA-181a-5p mimic  miRNA-181a-5p inhibitor | 1.29±0.13  1.44±0.54  1.53±0.32 |
|  | miRNA-23a-3p mimic | 0.95±0.02 |
| 72 hours after transfection | miRNA-23a-3p inhibitor  miRNA-181a-5p mimic  miRNA-181a-5p inhibitor | 0.84±0.27  1.33±0.09  1.21±0.07 |
